# Temporal dynamics of user activities: deep learning strategies and mathematical modeling for long-term and short-term profiling

**DOI:** 10.1038/s41598-024-64120-6

**Published:** 2024-06-24

**Authors:** Mohammed Kayed, Fatima Azzam, Hussien Ali, Abdelmgied Ali

**Affiliations:** 1 Faculty of Computers and Artificial Intelligence, Beni-Suef University, New Bani Sewif, Egypt; 2 Computer Science Department, Faculty of Science, Minia University, Minya, Egypt; 3 Computer Science Department, Faculty of Graduate Studies for Statistical Research, Cairo University, Giza, Egypt

**Keywords:** Mathematical modelling, Profiling, Social media, Multiclass classification, Deep learning, Computer science, Information technology, Computational science

## Abstract

Profiling social media users is an analytical approach to generate an extensive blueprint of user’s personal characteristics, which can be useful for a diverse range of applications, such as targeted marketing and personalized recommendations. Although social user profiling has gained substantial attention in recent years, effectively constructing a collaborative model that could describe long and short-term profiles is still challenging. In this paper, we will discuss the profiling problem from two perspectives; how to mathematically model and track user’s behavior over short and long periods and how to enhance the classification of user’s activities. Using mathematical equations, our model can define periods in which the user's interests abruptly changed. A dataset consisting of 30,000 tweets was built and manually annotated into 10 topic categories. Bi-LSTM and GRU models are applied to classify the user’s activities representing his interests, which then are utilized to create and model the dynamic profile. In addition, the effect of word embedding techniques and pre-trained classification models on the accuracy of the classification process is explored in this research.

## Introduction

The exponential growth of Online Social Networks (OSNs) has created a communicative and interactive phenomenon that allows billions of users to share their thoughts and communicate with one another in different ways. Through OSNs, everyone has the opportunity to create and share content that reflects their personality and interests, which can be changed over time according to several circumstances as they grow, experience new things, and interact with different events, influences, and environments. A user's profile on OSNs could be *static*, *dynamic*, or both. A *static profile* remains relatively unchanged over time. It contains basic information such as a bio, profile picture, and contact details. Updates are infrequent and primarily occur when there are significant changes or milestones. In contrast, the *dynamic* profile is automatically modified, adapted, and augmented to reflect the changes that occurred to the user’s nature and characteristics. Social media profiling is the process of gathering and analyzing information about individuals based on their activities, interests, demographics, or behaviors on social media platforms to build a detailed profile or persona that provides insights into a person's preferences, interests, and characteristics. Users’ behavior refers to the actions, interactions, and patterns exhibited by users within a system or platform. It encompasses a wide range of actions, including browsing behavior, engagement with content, preferences, reactions (such as likes, shares, comments), purchase behavior (in e-commerce contexts), and more. Users' behavior provides insights into their interests, preferences, intentions, and engagement levels. While users’ activities typically refer to specific actions or tasks performed by users within a system or platform. These actions can be more narrowly defined than users' behavior and may include specific events or operations such as posting, writing a comment, liking a post, sharing content, etc. Activities are often tracked and recorded as discrete events and can be analyzed to understand user interactions with the system or platform.

User profiling can be used for various purposes, such as marketing, targeted advertising, personalization, recommendation, and audience segmentation. Incorporating time as a factor in the process of building dynamic profiles varies between long and short term. *Short-term* profiles capture the current interests of the user. On the other hand, *long-term* ones reflect relatively stable interests and are not subject to frequent fluctuations over time. Due to some unusual and temporary events, like wars, crises, the World Cup, etc., different users with various and other interests may show an unprecedented interest in these topics, so studying the changes in the user’s interests and activities over long and short periods leads to better configuration of his dynamic profile. The distinction between long-term and short-term can vary depending on the specific application, domain, and objectives of the analysis. By constructing long-term profiles, researchers can gain insights into the user's overarching preferences and behaviors, allowing for personalized recommendations and tailored experiences over extended periods. For example, long-term profiles can track changes in a user's career trajectory, evolving hobbies, or shifting lifestyle preferences, enabling platforms to offer relevant content and services over time. On the other hand, short-term profiles capture more immediate shifts in user interests and activities, facilitating real-time adaptation and responsiveness. For instance, short-term profiles can reflect temporary interests, such as trending topics, current events, or seasonal preferences, allowing for timely recommendations and contextualized interactions. Together, the combination of long-term and short-term profiles provides a comprehensive understanding of the user's dynamic behavior, enhancing the effectiveness of personalized services and improving user satisfaction.

In this research, we are studying social user modeling and trying to answer the following research questions concerning the temporal change of a user’s profile inferred from his activities: (1) Can we adapt a model to describe both short- and long-term profiles? (2) How can we check the changes in the user's behavior during certain periods? (3) How can we improve the classification process used to classify users’ different activities for better profile construction? To answer these questions, we (1) Introduced how to use our mathematical model for creating long and short-term profiles for OSNs users, (2) Suggested a technique to track the changes in user behaviors, (3) Proposed two RNNs models to classify users’ activities, (4) Investigated the effect of combining pre-trained word embedding techniques (s.a FastText and GloVe) with RNNs models on classification accuracy, and finally (5) Tried to achieve better classification accuracy by fine-tunning BERT model as a classifier.

The remainder of this paper is organized as follows. Section “Related work” discusses some of the related works. Section “Proposed framework” provides our proposed approach. Section “Experimental results and discussion” describes the study’s experiments. Finally, Sect. “Conclusion and future work” concludes the study and proposes future research directions.

## Related work

This section presents a review of previous methodologies that discussed the problem of discovering users’ interests and building profiles. The reviewed literature is categorized into two groups: research centered around users' preferences and profiles and those centered around text classification.

### User’s interests and profiles

Many researchers have discussed user profiling (or user classification) on SMNs for various purposes and using different techniques. In their research^1^ presented a Behavior Factorization (BF) model for constructing topic interest profiles for social media users. They analyzed a large quantity of behavior data from users in Google+ and found that users' topic interests exhibited by one type of behavior are different from other types. To build the profile, the BF first learns a latent embedding model by factorizing matrices separated by behaviors, then builds user topic profiles for different types of behaviors using this embedding model. Dougnon et al.^2^ designed an algorithm called Partial Graph Profile Inference+ (PGPI+) to infer users’ profiles under a partial social graph constraint. The algorithm does not need training, and it offers the advantage of user control over the balance between the extent of gathered information for profile inference and the resulting inference accuracy. The algorithm has the advantage of using useful information like friendship links, user profiles, and group memberships, as well as the” likes” and” views” from social networks such as Facebook when available.

On-at et al.^3^ proposed a dynamic keyword-based user profile that represents his interests through numerical weights. They used the user's egocentric networks as sources to collect necessary information about his interests and to build his social profile. In order to achieve the dynamic concept and to reflect the evolution of users' interests, a scoring function is used with temporal criteria to weigh each extracted element and information of the user’s social networks. Farnadi et al.^4^ presented a hybrid deep learning user profiling framework based on both user’s generated content and their social relational content. It employs a common representation across modalities, facilitating the fusion of data from three distinct sources (visual, textual, and relational) at the feature level. At the decision level, the approach combines the resulting decisions from different networks that operate on each collection of data sources to obtain better profiling. Chen et al.^5^ developed a semi-supervised classification paradigm to predict a user's profile using a heterogeneous graph structure. In their heterogeneous graph attention networks (HGAT) model, the entities of interest (e.g., items, users, attributes of items, etc.) are represented as nodes, while the interactions between entities are the edges. The model learns the representation of each entity by considering the graph structure and then uses the attention mechanism to determine the relevance of each neighbor entity.

For influencer marketing,^6^ introduced a multimodal deep learning model that utilizes both text and image data of Instagram users' posts to classify both influencers and their individual posts into specific topics and interests (s.a., family and fitness). To the best generation of influencer representations, they identified the more relevant posts to the topics of influencers using the attention mechanism. De Campos et al.^7^ represented the users by hybridizing two different homogeneous sub-profiles (temporally and topically). To construct the topical sub-profiles, they used LDA (Latent Dirichlet Allocation) for performing a clustering process. The temporally sub-profiles are built by dividing the user's interactions into time intervals and computing the frequency of interactions within each interval. Finally, it combines both prior methods of profile construction by simultaneously leveraging the topical and temporal aspects in order to obtain consistent sub-profiles in terms of both traits. Table 1 shows a comparative analysis of the aforementioned research. It is important to acknowledge that the lack of standardized datasets and benchmarks makes it unfair to compare profiling methods directly. Furthermore, the variations in platforms, user demographics, profiling criteria, techniques, and evaluation methodologies across studies make a comprehensive and accurate comparison challenging. Our efforts have focused on evaluating user profiling methods across multiple tasks or settings to gain insights into the strengths and limitations of different profiling techniques. As a result, the comparison will be approximate in terms of evaluating criteria, results, and the strengths and weaknesses of each method.

**Table 1:** A rough comparison between profiling research.

Author(s)	Evaluation criteria	Results	Advantage(s)	Disadvantage(s)
^1^	The ability to build user profiles using the Direct Profile Building (DPB) method improve the coverage of user profiles on specific behavior types	When compared with the Baseline model using the Direct Profile Building (DPB) method, the BF model achieved an 89% improvement in Normalized Discounted Cumulative Gain NDCG, a 93% improvement in Average Percentile, and an 82% improvement in Recall The BF model showed about 100% improvement in the performance of building user's behavior-specific profiles CBSUP compared to the Baseline model The Weighted Profile Building (WPB) method improved user coverage by more than 70% for specific behavior types, such as Commenting and + 1	The method improves accuracy and cleanliness of topic interest signals by separately modeling users' interests from various behavioral signals, resulting in more precise topic predictions for different user behaviors The Weighted Profile Building (WPB) method enhances user coverage by considering different behavior types, especially for users without specific behavior types, while maintaining reasonable performance The method allows for flexibility in generating various types of user profiles based on specific behavior types making it adaptable for different content recommendation applications	Does not Support dynamic profiling The method is domain specificity as it may excel on social media platforms like Google+, where different behavior types signify distinct user interests, but may struggle to generalize to other domains lacking such clear distinctions Implementing the Behavior Factorization approach, especially at large scales in real-world systems, may require significant computational resources and expertise in matrix factorization techniques. This complexity could be a barrier to adoption in some applications
^2^	predicting attribute values of users in social networks	The three proposed models' accuracy for nominal attributes using the Facebook dataset is 96%, 84%, and 96% Prediction Accuracy for attributes such as status (student/professor) and gender can be predicted with more than 95% accuracy	It offers improved accuracy in predicting user profile attributes compared to existing algorithms, especially for numeric and nominal attributes Provide accurate predictions while accessing a smaller number of nodes from the social graph, making it more efficient in terms of data retrieval Introduces a mechanism to calculate certainty values for predictions, allowing users to gauge the reliability of inferred attributes	Attribute Complexity: Certain attributes, such as region, may be more challenging to predict accurately due to various factors, potentially requiring further refinement in prediction methods Prediction Variability: Some attributes of the same type may exhibit varying prediction accuracies, indicating potential challenges in consistently predicting certain attributes across different user profiles
^3^	The ability to build interest profile	Precision = 22.5% Recall = 12.18%	The model shows improvements in precision and recall compared to existing time-agnostic approaches Using a machine learning approach, the model can be customized and parameterized based on the characteristics of the targeted social network, allowing for flexibility and adaptability By incorporating time decay rates for relationships and information, the model can capture temporal dynamics and changes in user interests over time, addressing the cold start problem for inactive users	The model may face challenges in accurately matching keywords, especially when keywords are too specific or when there are synonyms, leading to potential inaccuracies in user interest extraction The model's effectiveness may vary across different types of social networks, with potential limitations in more general social networks like Twitter or Instagram, where user interests are diverse and dynamic The model's parameterization and time-awareness features may introduce complexity in implementation and maintenance, requiring careful tuning and monitoring to ensure optimal performance
^4^	Predicting user attributes like age, gender, and personality traits	Age prediction: Area Under Curve (AUC) score > 0.9 Gender prediction: Area Under Curve (AUC) score > 0.95	Superior performance in predicting user attributes such as age and gender compared to existing methods The model captures a more comprehensive view of user behavior and characteristics	The model's effectiveness may be dependent on the availability and quality of data from different modalities, which could pose challenges in real-world applications Deep learning models, especially those integrating multiple data modalities, may lack interpretability, making it challenging to understand the underlying reasons for specific predictions
^5^	Prediction of user attributes	Gender prediction: Accuracy = 57% Macro-F1 = 56% Age Prediction: Accuracy = 44% Macro-F1 = 24.6%	The frameworks effectively integrate various types of data in the network, leading to appealing performance in user profiling tasks The models automatically model multi-relation graph structures and node features in heterogeneous networks without the need for hand-crafted features or fusion methods The models can leverage unsupervised information along with limited user labels to construct predictors, enhancing the profiling process	The models may introduce complexity due to the incorporation of attention mechanisms and graph structures, which could make them harder to interpret and implement in some cases The effectiveness of the models may heavily rely on the quality and diversity of the input data, which could be a limitation in certain scenarios
^6^	Classifying influencers into their respective interest categories	Influencer Classification: Accuracy = 98% F1-score ≈ 89: 99% Post classification: Accuracy = 96% F1-score ≈ 89: 99%	Incorporating both text and image information from social media posts, the model captures a more comprehensive view of influencers' content, leading to more accurate profiling The use of the attention mechanism helps in selecting relevant posts for generating influencer representations, enhancing the model's performance	The quality and quantity of the data available for training may influence the model's performance, potentially leading to biases or limitations in the model's generalizability Social media trends and influencer behaviors evolve rapidly, making it challenging for static models to adapt and capture the latest patterns and preferences in influencer content
^7^	Using topical and temporal profiles to build subprofiles	MRR@40 = 0.3935 R@1 = 0.2501 R@5 = 0.5655	By combining topical and temporal dimensions, the model can provide more accurate and relevant recommendations for publication venues The model reflects the natural evolution of topics over time by grouping articles with similar underlying topics into temporal subprofiles. This feature can capture the dynamic nature of research fields and ensure that recommendations align with current trends and developments The weighting mechanism can enhance the relevance of recommendations	Building and maintaining topical and temporal profiles for a large collection of articles can impose computational overhead and storage requirements. Processing and updating these profiles in real time may be resource-intensive The model's performance may be sensitive to tuning parameters related to temporal decay, weighting of subprofiles, and fusion methods. Finding the optimal configuration for these parameters could be a nontrivial task

### Text classification

Classifying the user's generated content is an essential step in generating his dynamic profile. Many research papers discussed text classification problems and proposed different solutions. In their research,^8^ tried to enhance the accuracy and effectiveness of text classification by proposing a novel term weighting approach. They adopted an existing TextCNN model^9^ by combining the word embeddings with the new scheme of term weighting that takes into account the varying importance of terms in documents with different class labels. The scheme assigns multiple weights to every term so that each weight can appropriately reflect its importance to the documents coming from different text classes. For the multi-label classification task,^10^ presented a sequence-to-sequence (Seq2Seq) based learning model, which captures both local and global semantic information in text through its encoder and decoder modules. The encoder combines CNN and recurrent neural network (RNN) together to extract the local semantic features and capture long-range distance dependencies of features. The decoder, on the other hand, employs RNN to capture the global label correlation and also initialize a fully connected layer that reflects the correlation between any two different labels.

Xu et al.^11^ proposed a solution for data sparsity in a deep learning classification model for short text by utilizing a probabilistic knowledge base to represent words and sentences. Data sparsity refers to the fact that short texts often contain too few words to provide enough information for accurate classification, which affects the performance of the classification. They combined word embeddings and concept embeddings to enrich text representation and help the model utilize word-level knowledge instead of sentence-level. Li et al.^12^ suggested a recursive data-pruning solution for the misfitting problem in a CNN model used for text classification, which means that CNNs may capture irrelevant words in the dataset due to limited training samples and over-parameterization, which can lead to unsatisfactory performance in text classification tasks. Their solution started after standard training by evaluating all convolutional filters based on the discriminative power of generated features in the pooling layer. Subsequently, filters exhibiting lower evaluation scores are determined, and the words associated with these poorly performing filters are removed from the training data. This process is iterated to recursively eliminate the task's irrelevant words. Eventually, the cleaned data is used to train the single convolutional layer CNN model, which leads to better generalization.

To improve the performance of short text classification,^13^ explored the use of word taxonomies to construct semantic feature vectors that are used to enhance the feature vectors generated by traditional text processing algorithms such as tf-idf. Their tax2vec approach helps in exploring and understanding how the external semantic information could be incorporated into current (black box) machine learning algorithms, as well as revealing the nature of the acquired knowledge. Semantic features were also used by^14^ with a modified deep-learning model to improve the accuracy of short-text classification. They proposed an approach called CRFA (Context-Relevant Features with multi-stage Attention based on Temporal Convolutional Network (TCN) and CNN), which consists of 3 layers: embedding, representation, and output layer. To reduce short-text ambiguity and sparsity, they used an external knowledge base called "Probase” within the embedding layer to enhance the representation on both word and concept levels. The representation layer is composed of a two-level TCN-based attention model, WTCN (Word-level TCN) and CTCN (Concept-level TCN), to select discriminative concepts and word features for short text classification.

## Proposed framework

Our framework has two main axes: classifying the user’s activities and constructing his dynamic profile. The following subsections clarify each axis.

### User profile with temporal dynamics

Weighted-based user profile is a representation in which the user profile is represented by a keyword or a set of keywords that is directly provided by the system or automatically extracted from web pages or documents. Keywords are associated with numerical weights to represent the user's interests in different topics or categories.

In our previous research^15^, we considered a user *u* inside the social media group *ɡ*, with a static profile $P_{u}$ and discussing N topics. We used a weighted-based user profile to present the dynamic profile of the user. $D_{u} (t)$, which reflects the position $x_{u}$(*m*-dimensions) of the user inside the topic sphere such that $x_{u} (t_{i} ) = (d_{u}^{{c_{1} }} (t_{i} ),d_{u}^{{c_{2} }} (t_{i} ),...,d_{u}^{{c_{m} }} (t_{i} ))$. $d_{u}^{{c_{j} }} (t_{i} )$ is the distance between the user and the jth topic after the ith iteration is a representation in which the user profile is represented by a keyword or a set of keywords that is directly provided by the system or automatically extracted from web pages or documents. Keywords are associated with numerical weights representing the user's interests in different topics or categories.

Our model is based on the following assumptions about the connection between the user and topics:

1. The topics the user is interested in represent 100% of his mind.
2. The total similarity between the user and each topic depends on the user’s static profile $sim_{u}^{{c_{j} }} \left( {t_{0} } \right)$, the user's activities $A\_sim_{u}^{{c_{j} }} \left( t \right)$, and the user's following list $F\_sim_{u}^{{c_{j} }} \left( t \right)$.
3. The user's interests found in his static profile are used to calculate the initial similarity between the user and each topic $c_{j}$.
4. User’s activities like posts P, shares S, or likes L have different significance weights.
5. The similarities between the user and the topic increased as the distance between the user and the topic decreased.
6. The distance between the user and each topic changed after each activity.

Consider bloggers who use social media to display their daily activities and aren't interested in wars or disasters. One day, a catastrophe occurred in their country, so they used their social accounts to express their feelings and to support the victims, etc. Their user profiles should reflect the unusual reaction to the crisis as a short-term interest and the entertainment and other elder interests as long-term ones.

In this paper, we will introduce how to use our model to accommodate the short-term and long-term profiles.

#### **Definition 1**

(*Temporal user profile*) The temporal profile $D_{u} (time)$ of user *u* is the position $x_{u}$ of the user inside the topic sphere based on specific timespans.

1 $$ x_{u} (time) = (d_{u}^{{c_{1} }} (time),d_{u}^{{c_{2} }} (time),...,d_{u}^{{c_{m} }} (time)), $$

where $d_{u}^{{c_{j} }} (time)$ is the distance between the user and the jth topic category at the end of a given period. For the long-term profile, the beginning point of the user is the creation of the profile till the current moment. Accordingly, the initial values will be determined as mentioned in the 3rd point by using the user’s static profile. On the other hand, the beginning of the user in the short-term profile is the start of the specified period. Hence, the start values of $d_{u}^{{c_{j } }}$ will be the user’s dynamic profile at the beginning of the time span. Using the temporal-based profile, we can explore how the user profile evolves over time; for example, we could investigate if there are any variations between the user’s profile generated on weekends compared to his profile on weekdays, etc.

In order to measure the difference between the two profiles, we apply the Manhattan distance (also known as L1-distance) in vector representation:

2 $$ L_{1} \left( {x_{u} \left( {time_{y} } \right),x_{u} \left( {time_{z} } \right)} \right) = \mathop \sum \limits_{i} \left| {\,d_{u}^{{c_{i} }} \left( {time_{y} } \right) - d_{u}^{{c_{i} }} \left( {time_{z} } \right)} \right|\,,\,\,\,\,L_{1} \in \left[ {0..2} \right] $$

The higher the L_1_ value, the larger the disparity between the two profiles, and vice versa. Manhattan distance provides an overall measure of similarity or dissimilarity between the two profiles. As it calculates the distance between two points by summing the absolute differences in their coordinates, it is more robust to outliers and variations in individual dimensions (i.e., it does not specify which interests contribute more or less to the overall distance). To analyze the user's behavior and detect if there is any unexpected change in it, we will calculate the squared differences to obtain more detailed information about the differences between each corresponding distance in the two profiles.

3 $$ squared\,difference\,for \, d_{u}^{{c_{i} }} \, = \left( {d_{u}^{{c_{i} }} (time_{y} ) - d_{u}^{{c_{i} }} (time_{z} )} \right)^{2} $$

The squared difference is used to calculate the squared value of the difference between the corresponding coordinates of two points in a multidimensional space. It is useful when assessing the magnitude of change within specific categories, as it amplifies differences between values. The squared distance may be sensitive to outliers and can overemphasize large differences, so it's typically utilized at the category level rather than for overall profile changes. By setting specific thresholds or criteria, we can define significant differences in user behavior or discover unusual changes in user interests. For example, we might consider elements with squared differences above a certain threshold to reflect a significant change. Criteria such as when a user becomes interested in a topic for the first time and for how long he was interested in it could be an indicator of whether it is a temporary change or if it will be a lasting one.

### Text-topic classification

**Figure 1:**
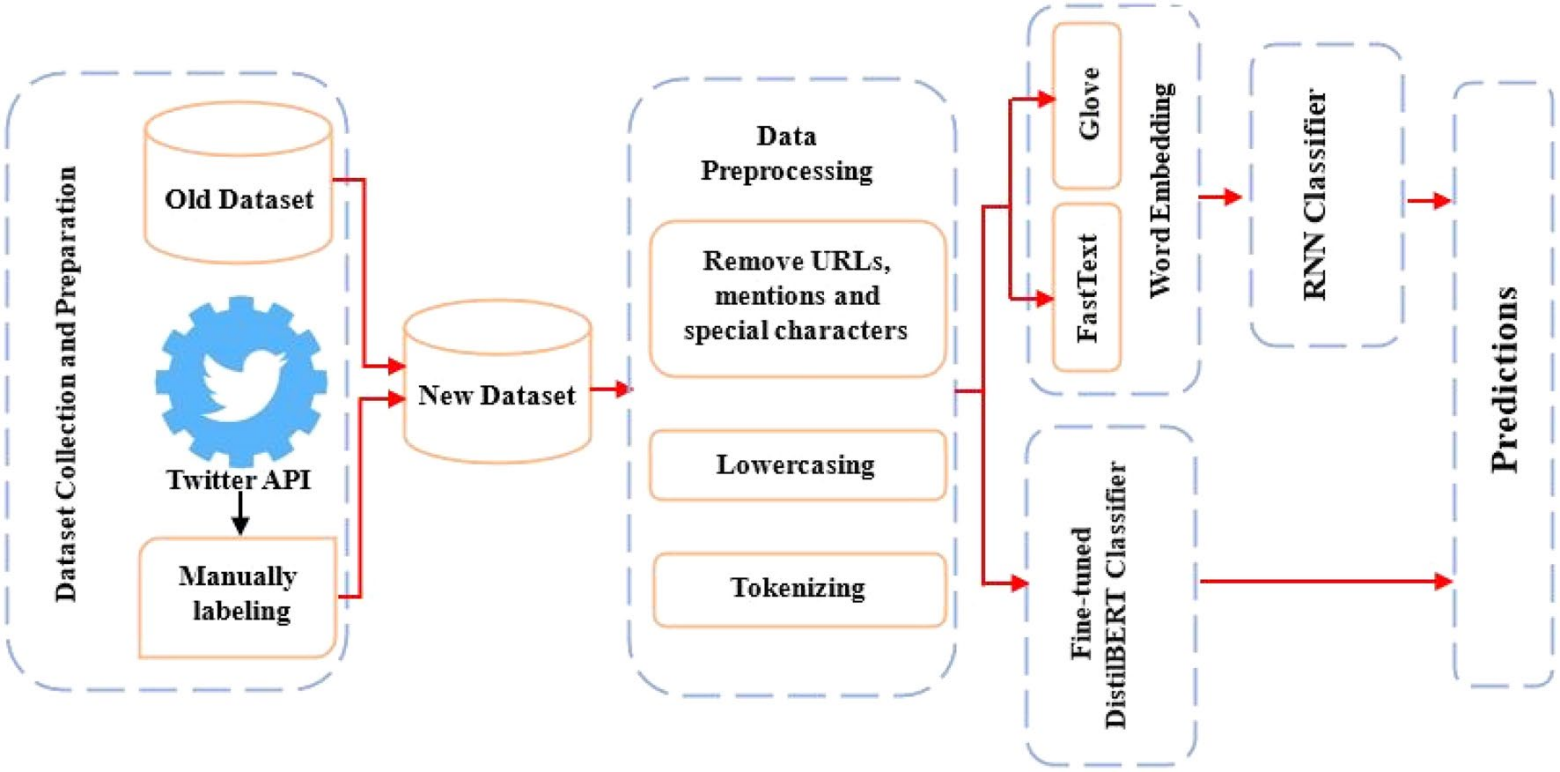
The architecture of proposed topic-classification models.

Classifying the activities of a user is a key task in creating his dynamic profile. Since deep learning models have consistently proven their effectiveness in resolving numerous text classification challenges, we used them to classify text into specific topics. Figure 1 shows an overview of the proposed models.

#### Data collection and preprocessing

We applied the models to two sets of tweets; the first one is the tweet data set collected by^16^, which consists of 22,424 manually labeled tweets divided into 11 topic categories (C1) business/finance, (C2) crisis [disaster/war], (C3) entertainment, (C4) politics, (C5) health/medical, (C6) law/crime, (C7) weather, (C8) life/society, (C9) sports, (C10) technology/internet, and (C11) others distributed as shown in Table 2. We observed that the dataset is imbalanced as there is a substantial disparity in the number of tweets between different classes, which could affect the performance of classifiers.

**Table 2:** The distribution of tweets among classes in the old dataset.

C1	C2	C3	C4	C5	C6	C7	C8	C9	C10	C11
1495	3288	3041	2963	311	944	565	2654	4104	1682	1377

In order to handle this problem, we modified the dataset in a way that each class contains 3500 tweets. For classes with tweets less than 3500, we collected relevant tweets using Twitter API to reach the specified number; on the other hand, classes with tweets more than 3500 are deducted by randomly removing redundant tweets. The final dataset consists of 35,000 tweets distributed equally between 10 categories by eliminating the ‘others’ class C11.

Preprocessing steps are applied to ensure that the tweets are clean and suitable for the classification process. We lowercase all tweets to eliminate case-related variations. Special characters except ($ and %), punctuations, URLs, mentions, and hashtags are removed. After that, we applied tweet tokenization by the tokenizer in the NLTK package.

#### Word embedding

After the tokenization, the tweet’s text is represented as vectors (numerical values) using an embedding model. Word embeddings are a type of distributed representation in an n-dimensional space designed to capture the semantic meanings of words. We used two distributed pre-trained word embedding models, GloVe^17^ and FastText^18^, to capture the semantic meaning of words in a sequence of text. Glove focuses on capturing global co-occurrence statistics of words in large text corpora, aiming to represent words based on their contextual relationships. In our model, we used GloVe embeddings that are trained on a large corpus with 300d vectors. FastText is an algorithm developed by Facebook that treats each word as a combination of n-gram characters, allowing it to represent out-of-vocabulary words and morphological variations effectively. FastText offers more flexibility and robustness in handling a wide range of languages and text types. We used FastText and GloVe separately and compared the results to study which one has a better impact on achieving higher classification accuracy.

#### Classification model

Embedding vectors produced by embedding models are fed into the deep-learning classification model. We applied two kinds of classification models in this paper:

1. Recurrent Neural Networks (RNNs): These are a type of neural network designed for processing sequential data. They have a unique ability to maintain an internal memory or hidden state that allows them to capture dependencies over time. However, traditional RNNs suffer from vanishing gradient problems during training, making it challenging to capture long-term dependencies effectively. To solve these issues, several modifications and variants of RNNs have been developed. Long Short-Term Memory (LSTM) networks^19^. introduce sophisticated gating mechanisms to control the flow of information, enabling them to capture long-range dependencies. Bidirectional LSTM (Bi-LSTM)^20^ processes data in both forward and backward directions, enhancing context understanding. Gated Recurrent Unit (GRU)^21^ is another variant of RNNs that is known for its efficiency and simplicity. They are effective at capturing sequential patterns and have been widely employed in various natural language processing tasks, text classification, and time series prediction, offering a balance between computational efficiency and modeling capability.
2. BERT Model: BERT^22^ is a transformer-based model that could be fine-tuned to solve a wide range of real-world NLP tasks. Fine-tuning BERT to classify text typically involves feeding labeled data to BERT and updating its parameters through backpropagation. This process allows BERT to leverage its pre-trained knowledge of language and semantics to excel in the classification task, often achieving state-of-the-art results with relatively little training data. In our experiments, we used a compact version of BERT called DistilBERT^23^ that is designed to be smaller and faster while maintaining much of BERT's language understanding capabilities. It achieves this by employing knowledge distillation techniques during training, where it learns from a larger pre-trained BERT model. The key distinctions lie in the reduced size and efficiency of DistilBERT, making it more suitable for applications with limited computational resources or a need for faster inference.

The first layer of the DistilBERT model involves the initial preprocessing and transformation of raw tweet text data into a structured format that can be fed into the DistilBERT model for further processing and classification. It encompasses tokenization, padding, truncation, the addition of special tokens to create input tensors, and creating attention masks. DistilBERT takes the tokenized tweet text as input and generates contextualized embeddings for each token in the text. These embeddings capture semantic and contextual information.

The model variant used for classification is “DistilBERT-base-uncased.” This variant is based on the DistilBERT architecture and is case-insensitive (lowercase). It is a smaller and more efficient version of the original BERT model. DistilBERT models typically consist of 6 layers of transformer encoder blocks, 768 hidden dimensions, and 12 attention heads in each multi-head self-attention mechanism. The vocabulary size of DistilBERT is typically 30,000. This means that the model can tokenize and work with a vocabulary of 30,000 unique sub-word pieces.

#### Evaluation metrics

The performance metrics used to evaluate our models are accuracy, precision, recall, and F1-score. Accuracy measures the overall correctness of the model's predictions by calculating the ratio of correctly classified instances to the total number of instances.

3 $$ Accuracy = \frac{Number\;of\;corrected\;topic\;predictions}{{Total\;number\;of\;predictions}} $$

Precision evaluates the model's ability to make accurate positive predictions within each class, indicating the fraction of correctly predicted positive instances among all instances predicted as positive.

4 $$ Precision = \frac{{Number\,of\;correct\;predictions\;of\;the\;topic \left( {TP} \right)}}{{Total\;number\;of\;instances\;predicted\;as\;that\;topic \left( {TP + FP} \right)}} $$

Recall, on the other hand, gauges the model's ability to capture all positive instances within each class, measuring the fraction of correctly predicted positive instances among all actual positive instances.

5 $$ Recall = \frac{{Number\;of\;correct\;predictions\;of\;the\;topic \left( {TP} \right)}}{{Total\;number\;of\;instances\;actually\,in\;that\;topic \left( {TP + FN} \right)}} $$

The F1-score is a balanced measure that combines precision and recall, providing a single value that reflects the model's overall performance across all classes.

6 $$ F1 - Score = 2 \times \frac{{\left( {precision \times recall} \right)}}{{\left( {precision + recall} \right)}} $$

Weighted average (WA) and macro average (MA) are two approaches for aggregating precision, recall, and F1-score metrics. Weighted average takes into account the class imbalance by assigning weights based on class proportions, giving more importance to the majority classes. This is useful when optimizing the model's performance with respect to class distribution. In contrast, macro average treats all classes equally, providing an unbiased assessment of the model's ability to perform across all classes, regardless of size or imbalance.

## Experimental results and discussion

### Text classification experiments

This section presents and discusses the experiments with the text-topic classification models. Our experiments are divided into three main dimensions: Studying the effect of the imbalanced dataset on the classification accuracy, studying the effect of feature extraction techniques, and the effect of using pre-trained models in the classification task. The datasets in all experiments are divided into two parts: 80% as the training set and 20% as the test set.

**Experiment 1:** In the first experiment, the Bi-LSTM and GRU models are applied to both the old and new datasets. Table 3 shows the significant change in performance across all metrics between the old and new datasets, showcasing the effectiveness of the updated dataset. This improvement in the performance between the old and new datasets suggests that the models have learned patterns that generalize better to unseen data.

**Table 3:** Comparison between Bi-LSTM and GRU models on the old and new datasets.

Performance metric	BI-LSTM		GRU	
	Old	New	Old	New
Accuracy	0.20	0.77	0.20	0.74
Macro avg				
Precision	0.10	0.78	0.10	0.74
Recall	0.12	0.78	0.13	0.74
F1-score	0.11	0.78	0.11	0.74
Weighted avg				
Precision	0.15	0.78	0.16	0.74
Recall	0.20	0.77	0.20	0.74
F1-score	0.17	0.77	0.17	0.74

**Experiment 2:** The second experiment is conducted to study the effect of different pre-trained word embeddings on the accuracy of classification using our new dataset. GloVe and FastText are used to construct the embedding matrix. This matrix serves as the initial weights for the embedding layer of our model. We chose a 300-dimensional vector to represent each word in the vocabulary, which passed to the next layer (Bi-LSTM or GRU). The models were trained for 50 epochs using the hyperparameters shown in Table 4.

**Table 4:** Hyperparameters used in Bi-LSTM and GRU models.

Parameter	Value	Parameter	Value
Input length	55	Cross-entropy loss	Categorical
Dropout	0.5	Batch size	16
Activation	Relu	Hidden dimensions	256
Optimizer	Adam	Output	SoftMax

In Table 5, the achieved results of the 2-models, along with pre-trained FastText and GloVe word embeddings, are illustrated. From the Table, we can see that (1) The Bi-LSTM model with FastText gives the best results, (2) The Bi-LSTM model achieves better results than GRU, and (3) FastText embeddings helped the models to achieve better accuracy.

**Table 5:** Comparison between Bi-LSTM and GRU models with FastText and GloVe word embeddings.

Performance metric	BI-LSTM		GRU	
	FastText	GloVe	FastText	GloVe
Accuracy	0.82	0.81	0.78	0.78
Precision	0.82	0.81	0.79	0.78
Recall	0.82	0.81	0.78	0.78
F1-Score	0.82	0.80	0.78	0.77

**Experiment 3:** The final experiment is conducted also on our new dataset to compare the performance of DistilBERT when it is fine-tuned as a classifier with the RNNs models’ performance. The key configurations of our model include a batch size of 128, a training duration of 50 epochs, the maximum sequence length for input text is set to 55, and the optimizer employed is Adam with a learning rate of 0.000001.

**Figure 2:**
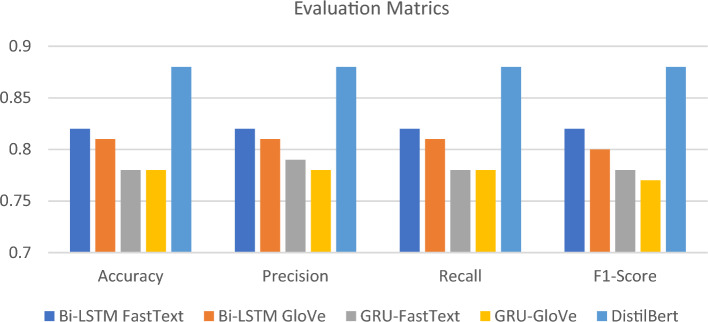
Comparison between all classification models.

The model achieves 0.88 accuracy, precision, recall, and F1-score, as shown in Table 6, which is better than previous RNN models, as shown in Fig. 2.

**Table 6:** The performance metrics of DistilBERT.

Performance Metric	Macro Avg
Accuracy	0.88
Precision	0.88
Recall	0.88
F1-score	0.88

**Figure 3:**
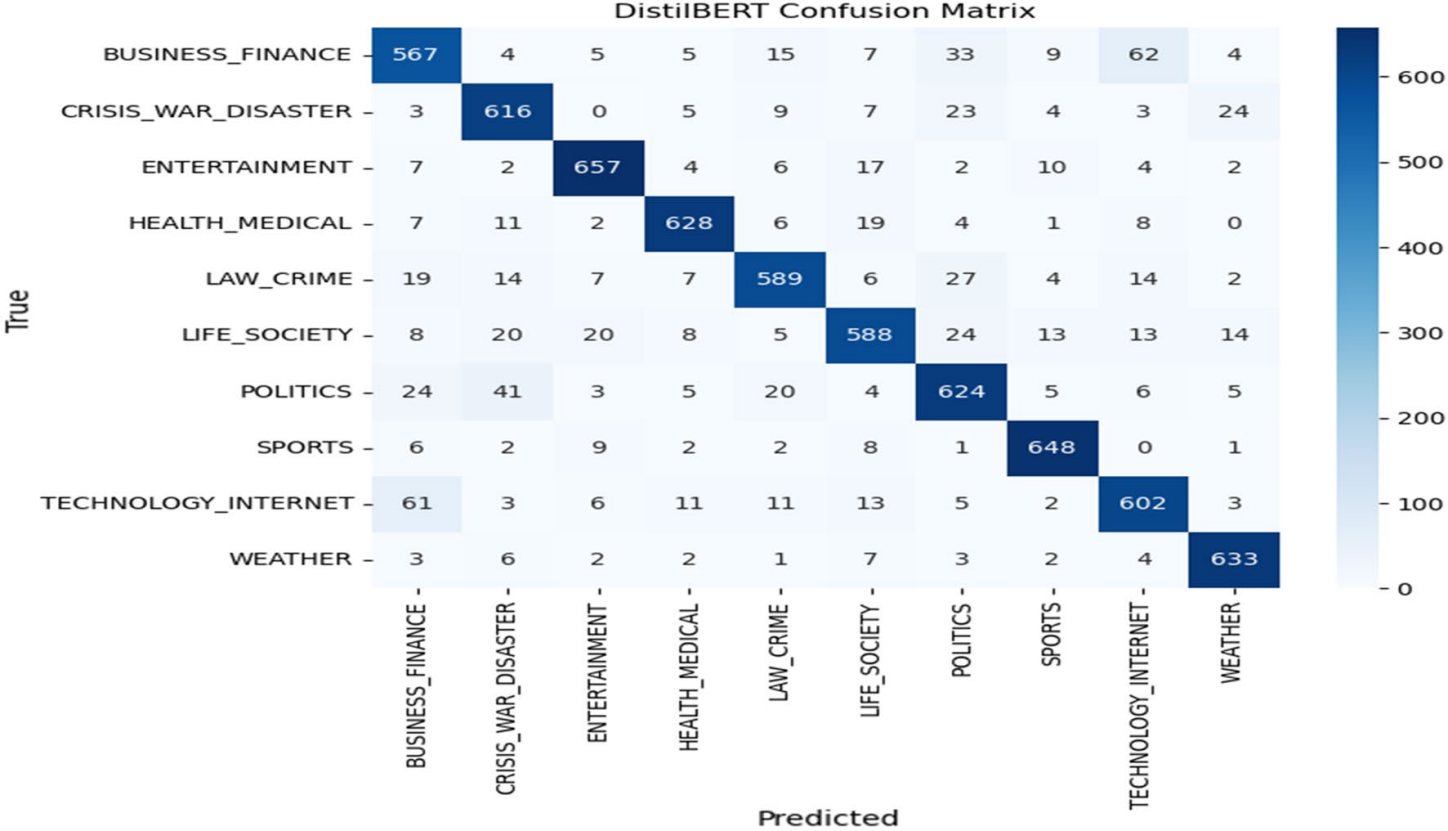
The confusion matrix of DistilBERT model.

For more analysis of the best model, Fig. 3 shows the confusion matrix, where the details of True positive (TP), False Positive (TP), True Negative (TP), and False Negative (TP) for each class are presented. We can notice that the “Business-Finance” class has many tweets that are classified as “Technology-Internet” and vice versa, which means that the instances of the two classes have similar features. Also, the “Politics” class has many tweets that are classified as “Crisis-War-Disaster”, and this may be due to the war tweets, which could have features similar to political ones.

### Statistical analysis

We deployed a non-parametric statistical hypothesis analysis, the Wilcoxon Signed Rank test, to statistically assess the difference between the proposed Bi-LSTM-FastText and DistilBERT models. This test is proposed by Frank Wilcoxon^24^ and popularized by Sidney Siegel^25^ and is to compare two matched samples, related samples, or to perform a paired difference test on repeated measurements of a single sample to determine if there are differences in their population mean ranks^26^. The null hypothesis assumes no difference between the population medians, while the alternative hypothesis suggests inequality. If the calculated p-value, which indicates the likelihood of chance differences, falls below the conventional significance level (usually 0.05), the test rejects the null hypothesis. Consequently, it is then deduced that a statistically significant difference exists between the two sets of samples, supporting the alternative hypothesis. The descriptive statistics shown in Table 7 show that the DistilBERT model has a higher mean accuracy (0.88) than the Bi-LSTM FastText model (0.82). The standard deviation is 0.00 for both models, indicating that there is no variability in the reported values. This suggests that all values for both models are identical.

**Table 7:** Descriptive statistics for the Bi-LSTM FastText and DistilBERT models.

Model	Mean	SD	Minimum	Maximum
Bi-LSTM FastText	0.8200	.00000	0.82	0.82
DistilBERT	0.8800	.00000	0.88	0.88

Table 8 provided Wilcoxon Signed-Rank Test results, which demonstrate a significant difference between the two models, “Bi-LSTM FastText” and “DistilBERT”. The Negative Ranks show that no negative ranks were observed, indicating that there were no instances where “Bi-LSTM FastText” performed better than “DistilBERT”. On the other hand, Positive Ranks show that DistilBERT performed better with 2.5 mean Ranks and a sum of ranks = 10.

**Table 8:** Summary of Wilcoxon signed-rank test results of the Bi-LSTM and DistilBERT models.

DistilBERT-Bi-LSTM FastText	Ranks		Test statistics	
	Mean ranks	Sum of ranks	Z-score	Asymp. Sig
Negative ranks	00	00	− 2.000-	0.046
Positive ranks	2.5	10.00		

Moreover, the statistical analysis revealed a significant contrast in accuracy between the two models, with a calculated Z-score of − 2.000 and a two-tailed *p* value of 0.046. The negative Z-value indicates that the accuracy of “Bi-LSTM FastText” is statistically significantly lower than that of "DistilBERT". The obtained *p* value of 0.046 implies that there is a mere 4.6% likelihood of observing such a substantial difference in accuracy between the two models by chance alone. Consequently, this difference is statistically significant at the conventional significance level of 0.05. These findings underscore the superior performance of “DistilBERT” over “Bi-LSTM FastText” in the evaluated context.

### Long and short-term profiling

In this section, we demonstrate how our model was used to determine the user's short-term and long-term profiles and positions over different time periods. Table 9 displays the changes in a user’s interests over time periods and how this change was reflected in both his long-term and long-term profiles. When creating the account, the user has specified his interests as entertainment (C3), life/society (C8), and sports (C9), so his initial position will be:

$$ x_{{u_{1} }} \left( {t_{0} } \right) = \left( {\infty ,\infty , 0.33, \infty ,\infty ,\infty ,\infty ,0.33, 0.33,\infty } \right) $$

**Table 9:**
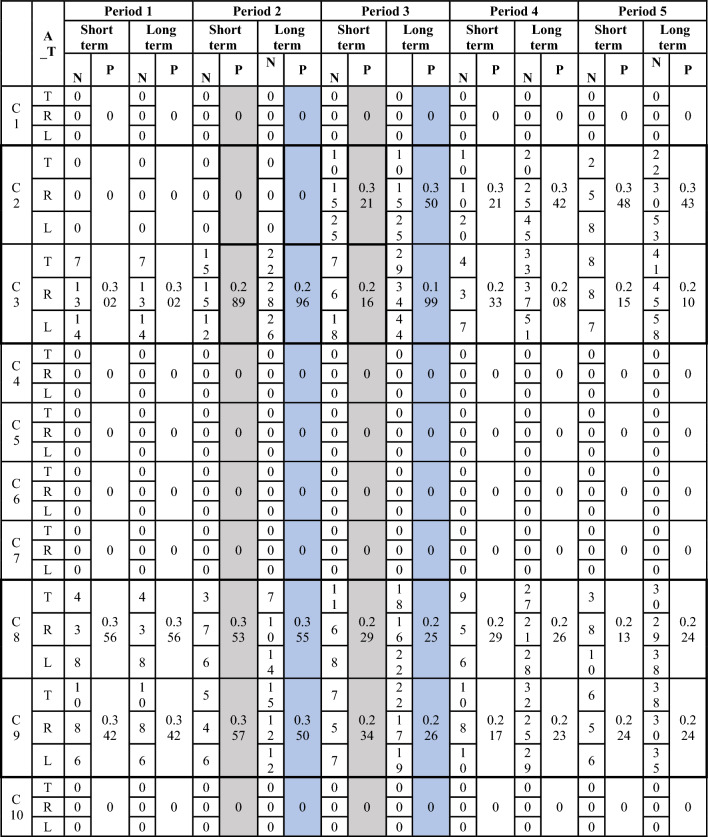
User’s short and long-term profiles and distribution of user’s activities (A_T: Activity type, T: number of tweets, R: number of retweets and L: number of Likes).

The user started to perform activities and his profile changed according to it. We took a snippet of his long and short-term profiles after five periods, and we noticed the following:

1. The user’s activities matched his interests in the first two periods.
2. After the second period, he suddenly started posting, liking, and sharing content related to the Crisis/War (C2) topic.
3. We applied Eqs. 2 and 3 to analyze the changes that occurred to his short profiles after periods 2 and 3.
  $$ x_{u} \left( {priod1} \right) = \left( {0,{ }0,{ }0.289,{ }0,{ }0,{ }0,{ }0,{ }0.353,{ }0.357,{ }0} \right) $$
  $$ x_{u} \left( {priod2} \right) = \left( {0,{ }0.321,{ }0.216,{ }0,{ }0,{ }0,{ }0,{ }0.229,{ }0.234,{ }0} \right) $$

- $$ \begin{aligned} & L_{1} \left( {x_{u} \left( {priod1} \right),x_{u} \left( {priod2} \right)} \right) = {\mid }0 - 0{\mid } + {\mid }0 - 0.321{\mid } + {\mid }0.289 - 0.216{\mid } + {\mid }0 - 0{\mid } \\ & \quad + {\mid }0 - 0{\mid } + {\mid }0 - 0{\mid } + {\mid }0 - 0{\mid } + {\mid }0.353 - 0.229{\mid } + {\mid }0.357 - 0.234{\mid } + {\mid }0 - 0{\mid }{ } \\ & \quad = { }0.321 + 0.073 + 0.124 + 0.123{ } = { }0.641. \\ \end{aligned} $$
- Squared Differences = [0, **0.103**, **0.005**, 0, 0, 0, 0, **0.0154**, **0.0150**, 0].

The results show a difference between the users' short profiles, especially in the second topic, which has a higher squared difference.

1. Similarly, we can apply these equations to long-term profiles to study the effect of the change.
2. The table also shows that the user’s interest in the new topic began to decrease gradually, which was reflected in the distance between the user and this topic, which began to increase again over time.

## Conclusion and future work

This paper presented a way to adapt our previous mathematical model to reflect both long- and short-term social profiles. Additionally, we can use the model to monitor changes in the user’s behavior during specific periods of time. Also, we proposed different classification models for classifying users’ activities used to construct their profiles. Moreover, in this research, we analyzed how the size of the dataset and unbalancing, word embedding techniques, and the use of pre-trained classification models affect the classification results. We built a tweets dataset by collecting and manually annotating tweets to achieve class balancing. Two pre-trained word embedding techniques, FastText and GloVe, are used separately with Bi-LSTM and GRU models to compare their impact on the classification accuracy. Finally, the DistilBERT model is applied in the downstream model to get better classification results.

For future work, there are many possible directions, including the following:

- Fine-tuned transformers in upstreaming models to generate features for classification models.
- Apply explainable AI techniques to understand influenced features in classification models.
- Increase the size of the dataset and try to exclude ambiguous tweets.
- Conducting an empirical analysis to investigate the influence of hyperparameters on the overall performance of topic classification models. This investigation may include examining hyperparameters like word embedding dimensions, the number of hidden units, and the selection of activation functions to determine their impact on model performance.

## Data Availability

The dataset used and analyzed during the current study is available from the corresponding author upon reasonable request.
